# Depletion of the *C. elegans* NAC Engages the Unfolded Protein Response, Resulting in Increased Chaperone Expression and Apoptosis

**DOI:** 10.1371/journal.pone.0044038

**Published:** 2012-09-05

**Authors:** Paul T. Arsenovic, Anthony T. Maldonado, Vaughn D. Colleluori, Tim A. Bloss

**Affiliations:** Department of Biology, James Madison University, Harrisonburg, Virginia, United States of America; Duke University Medical Center, United States of America

## Abstract

The nascent polypeptide-associated complex (NAC) is a highly conserved heterodimer important for metazoan development, but its molecular function is not well understood. Recent evidence suggests the NAC is a component of the cytosolic chaperone network that interacts with ribosomal complexes and their emerging nascent peptides, such that the loss of the NAC in chaperone-depleted cells results in an increase in misfolded protein stress. We tested whether the NAC functions similarly in *Caeonorhabditis (C.) elegans* and found that its homologous NAC subunits, i.e. ICD-1 and -2, have chaperone-like characteristics. Loss of the NAC appears to induce misfolded protein stress in the ER triggering the unfolded protein response (UPR). Depletion of the NAC altered the response to heat stress, and led to an up-regulation of *hsp-4*, a homologue of the human chaperone and ER stress sensor GRP78/BiP. Worms lacking both ICD-1 and the UPR transcription factor XBP-1 generated a higher proportion of defective embryos, showed increased embryonic apoptosis and had a diminished survival rate relative to ICD-1-depleted animals with an intact UPR. Up-regulation of *hsp-4* in NAC-depleted animals was specific to certain regions of the embryo; in embryos lacking ICD-1, the posterior region of the embryo showed strong up-regulation of *hsp-4,* while the anterior region did not. Furthermore, loss of ICD-1 produced prominent lysosomes in the gut region of adults and embryos putatively containing lipofuscins, lipid/protein aggregates associated with cellular aging. These results are the first set of evidence consistent with a role for *C. elegans* NAC in protein folding and localization during translation. Further, these findings confirm *C. elegans* as a valuable model for studying organismal and cell-type specific responses to misfolded protein stress.

## Introduction

To ensure survival, all organisms must tightly control the synthesis and folding of proteins. To this end, specialized proteins called chaperones regulate *de novo* protein-folding during and after translation, and recuperative protein-folding during denaturing stress. Disruption of protein folding underlies the pathologies of most, if not all, neurodegenerative disorders, including Alzheimer's, Huntington's, and Parkinson's diseases, as well as amyotrophic lateral sclerosis (ALS) [1], [2]. During disease development, the accumulation of improperly folded or aggregation-prone proteins leads to the formation of toxic oligomers that can trigger neuronal cell death [3]. To prevent protein aggregation, cells initiate stress responses to forestall the accumulation of misfolded proteins. These responses decrease translation, increase turnover, and manage existing proteins that might otherwise aggregate [4]. Misfolded protein management requires increased availability of chaperones, which inhibit aggregation through direct interaction with their targets [5].

Control of protein-folding begins at the ribosomal complex during translation. Highly conserved chaperones, known as the translational chaperone system, interact with both the ribosomal complex and the nascent polypeptide as it emerges. By doing so, chaperones prevent inappropriate associations between secondary structures and allow for the formation of functional domains as the peptide is released into the cytosol. Translational chaperone interaction may also be important for protein localization, specifically to organelles such as the ER and mitochondria. The translational chaperone system is best understood in *Saccharomyces cerevisiae*, where the heat shock protein (HSP) 70/40 triad complex and the NAC associate with growing peptide chains at the ribosome [6].

The HSP 70/40 triad is a complex of three chaperones, two of which form the ribosome-associated complex (RAC) that binds directly to the ribosomal complex, while the third, Ssb, binds both the ribosomal complex and the nascent peptide chain [7]. The NAC consists of two subunits, α and β, both of which bind to the polypeptide; βNAC also binds to the ribosomal complex [8], [9]. Previous studies in yeast associate the NAC with promoting protein localization away from the ER and to the mitochondria during translation, while more recent evidence suggests the NAC is also functionally connected to the HSP 70/40 chaperone network and plays an important role in proper protein folding [10], [11]. Deletion of NAC in Ssb-deficient yeast results in significant growth defects and accumulation of misfolded protein [12].

Although the NAC and the RAC are conserved throughout eukaryotic evolution, it is not clear that their chaperone functions in yeast are conserved in more complex organisms [12]–[14]. Our studies examine whether the NAC functions as a chaperone in *C. elegans*, which is genetically tractable much like yeast, but substantially more complex with regards to stress response. For example, eukaryotes respond to the accumulation of misfolded protein in the ER with the unfolded protein response (UPR), a mechanism that engages pathways involved in managing misfolded protein stress. In yeast, the UPR is mediated by a single transmembrane ER protein, Ire-1. However, *C. elegans* contains three transmembrane ER proteins, Ire-1, ATF6, and PERK/PEK-1, which independently or cooperatively initiate the UPR, and have conserved mammalian homologues [15]. Activation of these misfolded protein sensors can initiate a range of responses to maintain cell viability and functionality until acute stress is resolved and proteins are refolded properly. These responses include the attenuation of protein translation, stimulation of protein degradation, increase in ER and Golgi biogenesis, and up-regulation of chaperone expression [16]. If misfolded protein stress overwhelms the cell's ability to manage it, the UPR engages apoptotic pathways, which will kill the cell, or autophagic pathways, which can also lead to cell death. The ability of the UPR to kill chronically stressed cells links it to disease pathology, specifically proteopathic neurodegenerative diseases [17]. With this in mind, the role of the NAC in stress response is particularly interesting in *C. elegans*, a model organism with a well-defined and precisely lineaged nervous system that allows for the study of NAC chaperone function in neurons [18]. Induction of protein folding stress via depletion of the NAC in *C. elegans* may recapitulate aspects of neurodegenerative pathologies, establishing this particular experimental strategy as a viable model for stress-related neurodegeneration [19], [20].

The βNAC homologue ICD-1 is the best understood of the two NAC subunits in *C. elegans*. Deletion of ICD-1 results in inappropriate cell death throughout embryogenesis, with neurons more sensitive to removal of ICD-1 than other cell types [21]. In addition, depletion of ICD-1 also induces severe morphological defects throughout embryogenesis independent of the cell death phenotype, and *icd-1* knockout mutations are embryonic lethal. The α subunit of the NAC in *C. elegans* has not been characterized outside its predicted association with ICD-1. We propose to call this subunit ICD-2.

To determine the role the NAC plays in proteostasis and stress response in *C. elegans*, we depleted ICD-1 and -2 over time using RNA interference, and measured specific stress response outputs. We found depletion of the NAC modulated responses to heat stress and up-regulated the ER chaperone gene *hsp-4*, a homologue of mammalian GRP78/BiP [22]. Worms with a partially defective Ire-1 pathway were significantly more sensitive to *icd-1*(RNAi) effects than wild-type animals, including increases in apoptosis and mortality. Worms deficient in *hsf-1*, a cytoplasmic stress responder, were also more sensitive to ICD-1-depletion than wild-type, but still able to mount a stress response to heat. Up-regulation of *hsp-4* appeared specific to particular regions of *icd-1*(RNAi) embryos, with high levels of expression in regions containing gut and epithelial cells, and low levels of expression in neuronal regions. We also observed an increase in large lysosomal structures in the gut region of the animal possibly containing lipofsucin granules. Lipofuscins, also known as “age pigment”, are aggregates of cross-linked protein and lipids found in lysosomes and theorized to be the result of autophagy associated with postmitotic cellular aging [23].

## Results

### Depletion of the NAC modulates stress response, mortality and movement in the presence of heat

**Figure 1:**
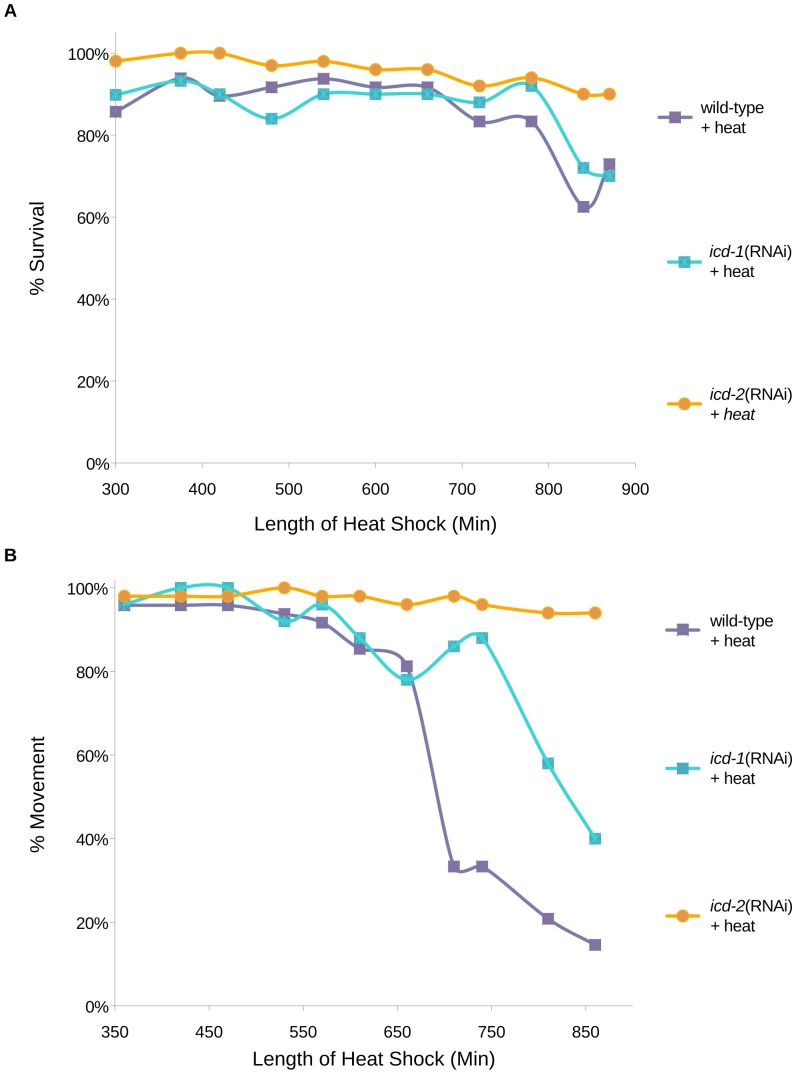
Mortality and movement of ICD-1 or ICD-2-depleted animals during heat stress. Developmentally synchronized wild-type (N2) *C. elegans* larvae were fed *icd-1* or *icd-2*(RNAi)-specific bacteria for 30 hours and exposed to continuous heat stress at 36°C for 850 minutes. A) Survival of animals was measured by periodic assessment of pharyngeal pumping. B) Movement was measured by periodic assessment of escape reaction to physical prodding. N = 50 for each of the three populations tested per experiment. Data presented in A) and B) represent the results of two independent trials for each experiment. The three experimental populations were also exposed to no heat (20°C) as a measure of basal rates of mortality and effects on movement, and showed no inherent lethality or immobility (N = 50 per population) (data not shown).

To characterize the putative chaperone role of the NAC, we examined the ability of NAC-depleted animals to cope with protein-denaturing heat stress. We reasoned that if the NAC is a component of a translational chaperone system, removal of either NAC subunit (ICD-1 or -2) would exacerbate misfolded protein accumulation during heat stress, and decrease worm survival. Synchronized worms treated with *icd-1* or *icd-2*(RNAi) were exposed to chronic heat stress, and survival was measured by the presence of pharyngeal pumping. Unexpectedly, animals depleted of ICD-1 or -2 were not more sensitive to heat stress relative to wild-type controls, as measured by mortality rates (Figure 1A). ICD-2-depleted worms were instead more resistant to death than either ICD-1-depleted or wild-type animals. To determine if the loss of the NAC affected the vitality of worms under heat stress, the movement of NAC(−) worms was measured relative to wild-type in the presence of heat. Animals with decreased levels of ICD-1 or -2 were more mobile in these assays, with differences in robustness; worms lacking ICD-2 showed the greatest movement, followed by ICD-1-deficient and wild-type animals (Figure 1B). Movement was strictly defined as an escape response from the touch of a metal pick, but in many cases, *icd-1* and *icd-2*(RNAi)-treated worms were quite functional and often moving and feeding in the absence of prodding, while their wild-type counterparts were immobile. Taken together, these results indicate that depletion of the NAC through RNAi treatment did not increase mortality in post-embryonic animals and positively affected movement in the presence of heat stress. One possible explanation for the increased vitality in NAC(−) worms under heat stress could be the induction of a compensatory stress response.

### ER chaperone *hsp-4* is up-regulated in the absence of ICD-1

**Figure 2:**
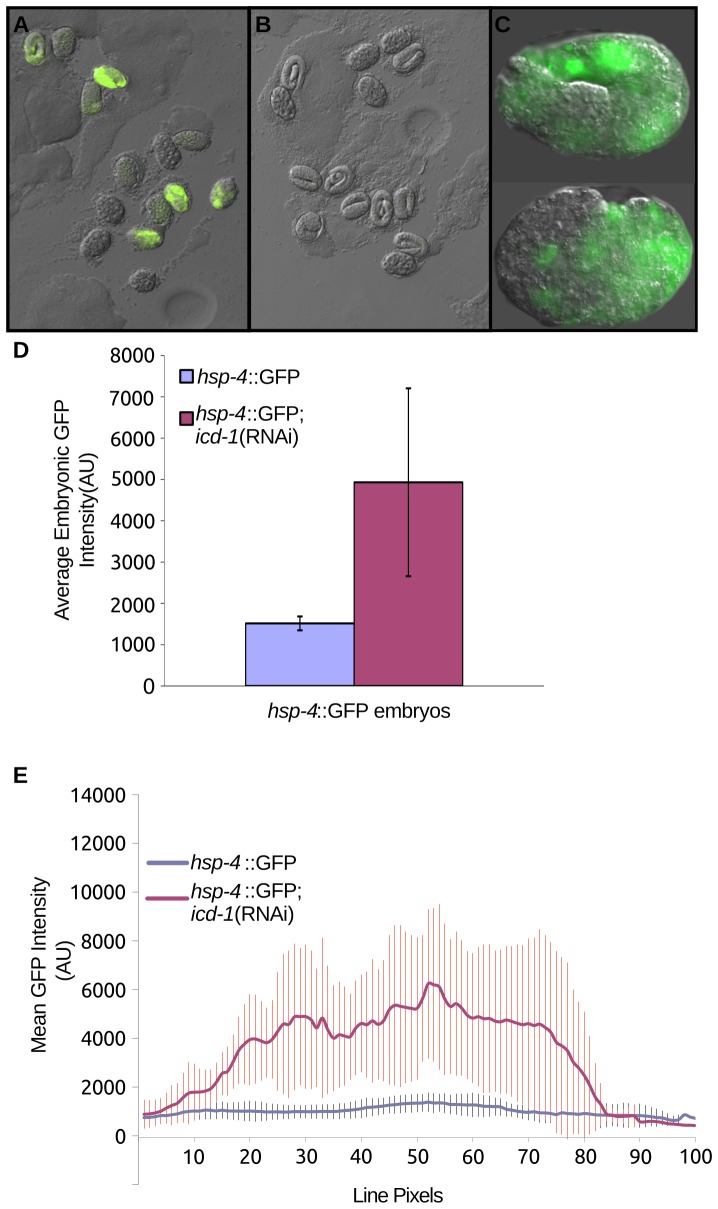
Embryonic expression of *hsp-4* in ICD-1-depleted animals. Animals containing an *hsp-4*::GFP expression vector were fed (A) *icd-1*(RNAi)-specific bacteria or (B) OP50 (*E. coli*) bacteria expressing no double stranded RNA for 36 hours and their progeny embryos were randomly assessed for expression of GFP. C) Two examples of embryos selected from the *icd-1*(RNAi) population. Both embryos were viable and morphologically wild-type. D) Average GFP signal of *hsp-4*::GFP-containing embryos treated with *icd-1*(RNAi) compared with GFP signal generated in control *hsp-4*::GFP-containing embryos. Populations of embryos were chosen at random, and embryos within a given field of observation were assessed and averaged regardless of GFP status. Up-regulation of *hsp-4::*GFP was observed in three independent experiments, and up-regulation was quantified in two of these experiments. Error bars represent the standard deviation of the mean GFP intensity of the embryos assessed in these two experiments (two-tailed t-test, P-value = 0.01). E) Averaged line scans measuring pixel intensity across all assessed *icd-1*(RNAi) embryos compared with control embryos; vertical bars represent the standard deviation of line pixels.

**Figure 3:**
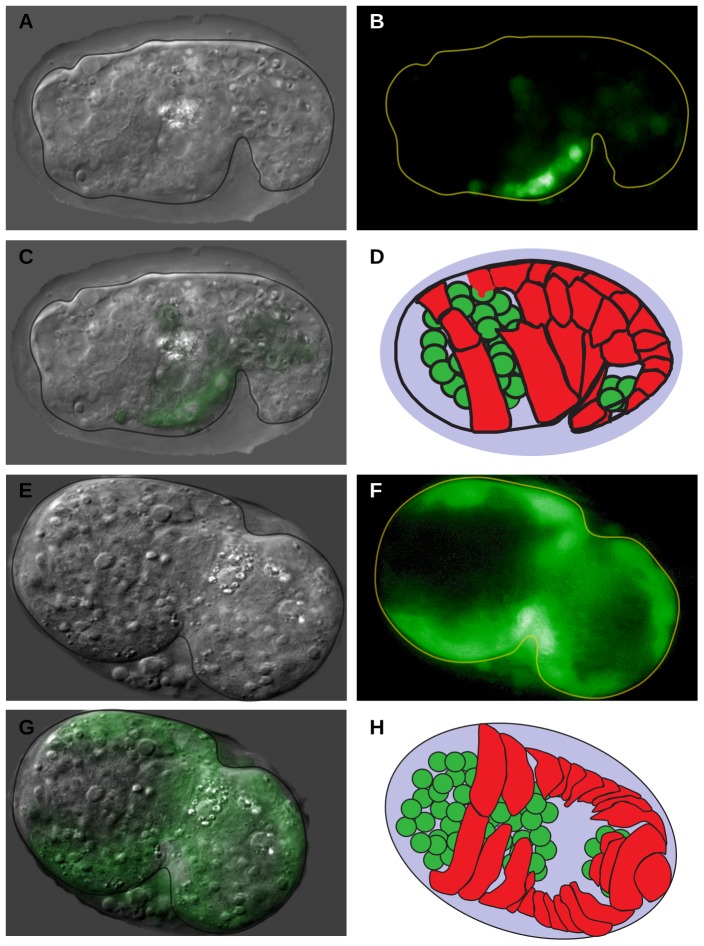
Localization of *hsp-4* expression in embryos depleted of ICD-1. Animals containing an *hsp-4*::GFP expression vector were treated with *icd-1*(RNAi) for 48–56 hours, and their embryos were assessed for GFP expression. Localization of *hsp-4*::GFP signal was determined in three independent experiments, totaling 30 GFP-positive embryos assessed. All embryos showed similar localization patterns. In this time period of the RNAi treatment, a majority of embryos are morphologically defective with high levels of cell death. A–C) DIC, GFP and merged images, respectively, of a representative *icd-1*(RNAi) embryo ∼470 minutes post fertilization. D) Diagram of embryo 470 minutes post fertilization. E–G) DIC, GFP and merged images, respectively, of a representative *icd-1*(RNAi) embryo ∼360 minutes post-fertilization. H) Diagram of embryo 360 minutes post fertilization. In both D) and H) green cells represent location of neurons, red cells represent location of developing epithelial layer. *Diagrams in D) and H) adapted from Chin-sang and Chisholm, 2000* [*45*].

To determine if depletion of the NAC up-regulates chaperone expression, we examined the expression of chaperones in the embryos of *icd-1*(RNAi)-treated adult worms. *icd-1*(RNAi) has been shown to generate strong phenotypes throughout embryogenesis in the progeny of treated adults, ranging from increases in apoptosis to severe morphological defects and embryonic lethality [21]. If loss of ICD-1 is up-regulating chaperone expression, we might expect to see this effect during embryogenesis. Four separate *hsp*::GFP reporter strains were depleted of ICD-1 over time, and expression patterns were determined throughout embryogenesis. *hsp-16*, a cytosolic chaperone, and *hsp-6* and -*60*, both mitochondrial-specific chaperones, showed no detectible increases in expression (data not shown) [24], [25]. *hsp-4*::GFP, an ER-specific chaperone, showed dramatic up-regulation, first detected at mid-embryogenesis, and increasing throughout embryonic development (Figure 2A–C). GFP levels were consistently higher in embryos treated with *icd-1*(RNAi) relative to untreated controls (Figure 2D) and *unc-22*(RNAi) controls (data not shown), as measured by the signal generated by the whole embryo, or the average level of expression at different points throughout the embryo (Figure 2E). Within embryos, *hsp-4* expression patterns were consistently localized to specific regions of the embryo. Regions of the embryo that contain gut and epithelial cells showed high levels of expression relative to regions that contain primarily neurons; these regions showed little to no increased expression (Figure 3A–H). Many of these embryos displayed phenotypes consistent with previous *icd-1*(RNAi) results: severe morphological defects and increases in cell death and degeneration (Figure 3A, E) [21].

### Animals lacking XBP-1 show increased sensitivity to loss of ICD-1

Up-regulation of *hsp-4* in the absence of ICD-1 is consistent with an increase in misfolded protein in the ER and the initiation of the UPR. HSP-4 is a *C. elegans* homologue of GRP78/BiP, an ER-localized chaperone and sensor of misfolded protein responsible for initiating the UPR. To determine the putative role of the UPR in response to loss of the NAC, we performed *icd-1*(RNAi) assays in *xbp-1* knockout (ko) animals. XBP-1 is a downstream effector of Ire-1, a UPR component regulated by GRP78/BiP [26], [27]. Ire-1 pathways are of particular interest because they initiate stress responses closely associated with *icd-1*(RNAi)-induced phenotypes, such as the up-regulation of chaperone expression and the initiation of apoptosis. If increased expression of *hsp-4* in *icd-1*(RNAi) is associated with an Ire-1-dependent stress response, we hypothesized that *xbp-1*(ko) animals would be more sensitive to the depletion of ICD-1 due to a deficient UPR.

**Figure 4:**
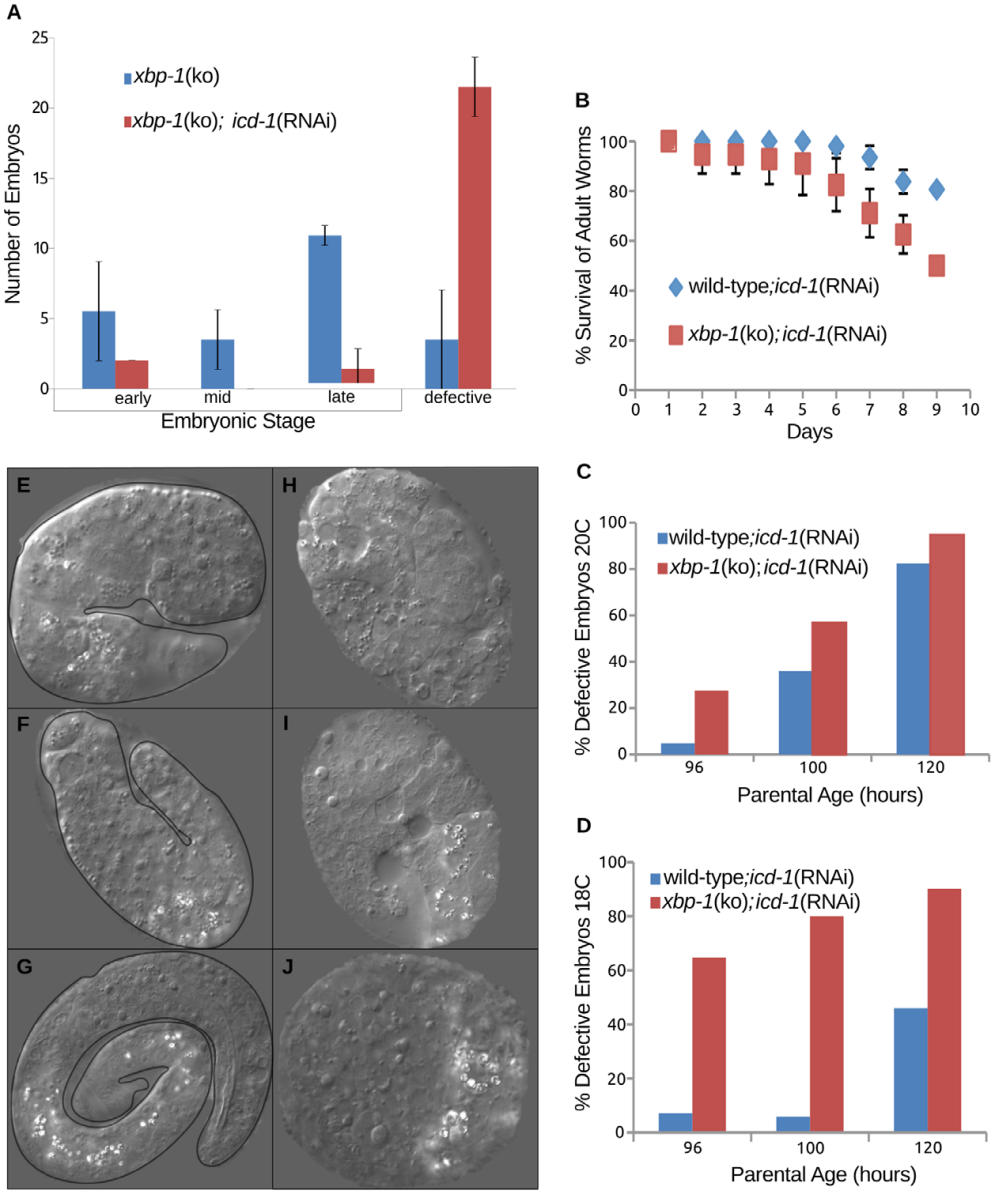
Increased sensitivity of *xbp-1*(ko) animals to depletion of ICD-1. [*hsp-4*::GFP] *xbp-1*(ko) worms were treated with *icd-1*(RNAi) and characterized for effects on mortality and embryonic development. A) Profile of embryonic development in [*hsp-4*::GFP] *xbp-1*(ko) animals untreated or treated with *icd-1*(RNAi). Embryos were scored as either phenotypically wild-type or defective. The stage of wild-type embryos was determined primarily by the extent of the vermiform structure, e.g. early embryos showed no tube-like formation, while late embryos contained fully formed worms. Defective embryos were morphologically abnormal with high levels of cell death. Error bars represent one standard deviation from the mean frequency of embryos per developmental stage over two independent experiments. B) Survival curve of [*hsp-4*::GFP] *xbp-1*(ko) and wild-type animals during *icd-1*(RNAi) treatment over a ten day period; survival was determined by the presence of pharyngeal pumping. Error bars represent the standard deviation from the mean of two independent experiments. C,D) Percentage of embryos characterized as defective in wild-type and [*hsp-4*::GFP] *xbp-1*(ko) animals treated with *icd-1*(RNAi) for 48–56 hours grown at 20°C (C) or 18°C (D) over two independent experiments. E–G) Representative defective embryos from wild-type adults treated with *icd-1*(RNAi) for 48 hours. All three embryos displayed moderate morphological defects with increased levels of cell death, but also display vermiform structure to some extent. H–J) Representative defective embryos from [*hsp-4*::GFP] *xbp-1*(ko) embryos treated with *icd- 1*(RNAi) for 48 hours. All three embryos showed severe morphological defects and significant cell death, with no detectable tube-like formations.

**Figure 5:**
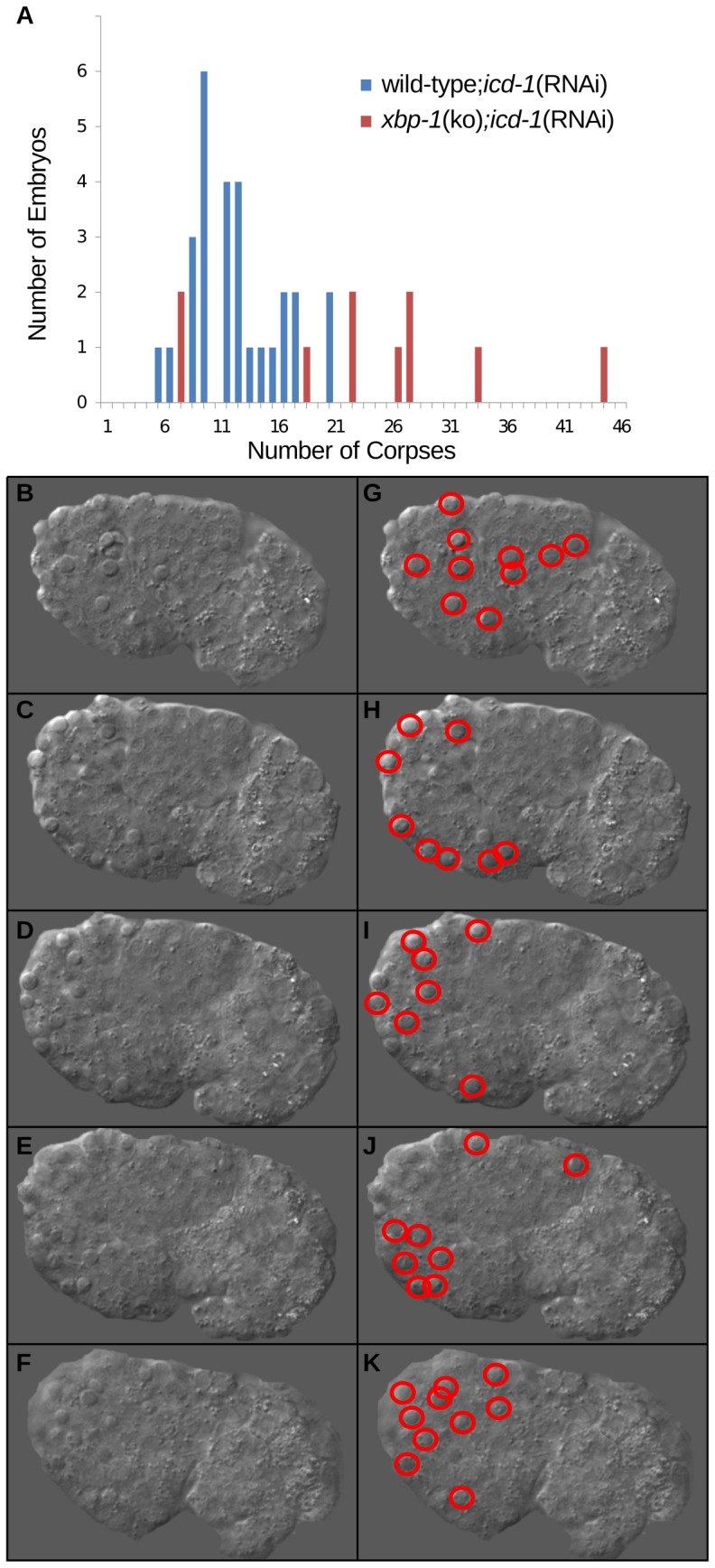
Embryonic apoptosis in *xbp-1*(ko) animals depleted of ICD-1. [*hsp-4*::GFP] *xbp-1*(ko) animals were treated with *icd-1*(RNAi), and their comma-stage embryos were scored for apoptotic cell corpses. Developmental apoptosis occurs normally throughout *C. elegans* embryogenesis, and the comma-stage of development generates a high number of apoptotic cell corpses relative to other stages. A) The number of comma-stage embryos containing a specific number of cell corpses in wild-type (n = 28) and [*hsp-4*::GFP] *xbp-1*(ko) (n = 11) embryos treated with *icd-1*(RNAi). B–F) Multiple focal planes of a comma-stage embryo produced by an [*hsp-4*::GFP] *xbp-1*(ko) worm treated with *icd-1*(RNAi). Cell corpses are distinguishable from surrounding cells by their raised-up, button-like structures. G–K) Duplicate images of A–E highlighting each of the 43 cell corpses observed in this embryo.

To determine the effects of ICD-1-depletion in *xbp-1*(ko) animals versus wild-type, we characterized the basal rate of defective embryo generation in *xbp-1*(ko) worms. This rate was significant, but still much lower than the rate of defective embryo generation in *xbp-1*(ko);*icd-1*(RNAi) animals (Figure 4A). When compared to wild type;*icd-1*(RNAi) animals at 20°C, *xbp-1*(ko);*icd-1*(RNAi) had a higher rate of mortality, with a concomitant increase in morphological defects and cell death in the embryos of surviving worms (Figure 4B,C). The rate of defective embryo generation in *xbp-1*(ko) worms depleted of ICD-1 increased dramatically when the temperature was dropped to 18°C (Figure 4D), indicating that other elements of the UPR may still be engaged in response to the loss of the NAC, and this response is acutely temperature sensitive. The morphology of the defective embryos in the presence and absence of XBP-1 also differed significantly; wild type;*icd-1*(RNAi) embryos showed morphological defects and cell death, as expected, but had a higher percentage with a discernible worm-like form (Figure 4E–G) relative to *xbp-1*(ko);*icd-1*(RNAi) embryos, which generally showed no worm-like form, and were often too deformed to stage developmentally (Figure 4H–J). The small percentage of *xbp-1*(ko);*icd-1*(RNAi) embryos that could be staged showed an increased sensitivity to early embryonic apoptosis relative to wild-type;*icd-1*(RNAi) populations; 22.3+/−11 cell corpses (n = 10) versus 11.75+/−3.9 cell corpses (n = 28) respectively (Figure 5A). In previous ICD-1 studies, control RNAi-treated worms displayed 6.3+/−2.3 cell corpses per embryo [21]. Corpse numbers in *xbp-1*(ko);*icd-1*(RNAi) embryos at the comma stage of development ranged from 6 to 43, and were generally localized to regions of the neuronal cell lineage, consistent with apoptotic effects seen in previous ICD-1 studies (Figure 5B–K) [21]. We also observed an increase in corpse-like structures and enlarged gut granules late in embryogenesis. These cell deaths were again found in the neuronal cell lineage, but also throughout the embryo, including hypodermal and gut cell regions, indicating that many cell types in *xbp-1*(ko) animals were sensitive to the ICD-1-depletion.

*xbp-1*(ko) mutants may be more sensitive to loss of the NAC due, in part, to their inability to increase chaperone expression in response to Ire-1 activation. Previous studies have shown that *hsp-4* expression is dramatically reduced in *xbp-1*(ko) worms relative to wild-type after treatment with tunicamycin, an ER-stress inducer [28]. In accordance, we found a less than two-fold increase in *hsp-4*::GFP expression in *xbp-1*(ko); *icd-1*(RNAi)-treated worms (Figure S1A,B), as compared with an almost five-fold increase in wt:*icd-1*(RNAi) animals, when both were compared to untreated controls (Figure 2D), indicating that *xbp-1*(ko) worms up-regulate *hsp-4* expression less efficiently than wild-type animals when both are depleted of ICD-1.

### Movement of ICD-1-depleted animals is partially dependent on HSF-1

**Figure 6:**
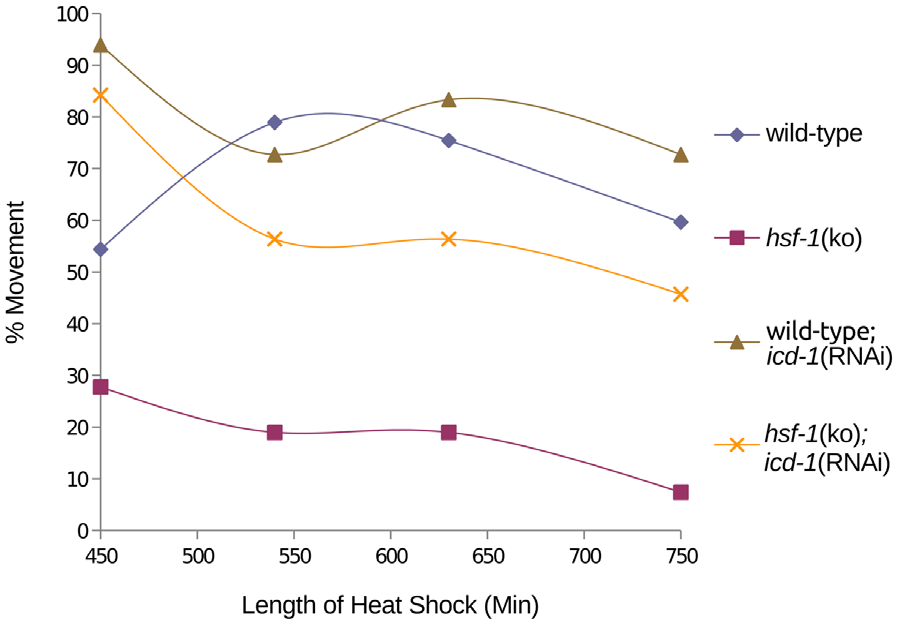
Effects of ICD-1 depletion on movement of *hsf-1*(ko) animals exposed to heat stress. *hsf-1*(ko) animals were fed *icd-1*(RNAi)-expressing bacteria at time 0 and exposed to heat stress at 36°C for 750 minutes. Movement of worms was first measured at 450 minutes of heat stress by escape response to prodding, and compared to wild-type;*icd-1*(RNAi) animals under heat stress (n = 400 for each population). All experimental populations showed 100% movement at the beginning of the time course (data not shown). As a control, both strains of worms were assessed for movement at 20°C (n = 400) (data not shown).

Accumulation of misfolded protein in the ER in the absence of the NAC is consistent with NAC's proposed role in modulating the interaction of the signal recognition particle (SRP) with nascent polypeptides, preventing inappropriate localization of proteins to the ER [11]. To determine if other compartments of the cell experience misfolded protein stress triggered by the loss of the NAC, *icd-1*(RNAi) was performed in an *hsf-1* knockout strain under heat stress. HSF-1 (heat shock factor −1) is a cytosolic detector of misfolded protein stress and well-characterized transcription factor for cytosolic chaperones [29]. If cytosolic chaperone expression contributes to heat stress resistance upon depletion of the NAC, *hsf-1*(ko);*icd-1*(RNAi) animals should show increased sensitivity to heat stress relative to wild-type; *icd-1*(RNAi) worms. We found this to be true; *hsf-1*(ko); *icd-1*(RNAi) worms were less mobile relative to wild-type;*icd-1*(RNAi) populations when both were stressed with heat (Figure 6). However, *hsf-1*(ko);*icd-1*(RNAi) do maintain movement at a strikingly higher rate than *hsf-1*(ko) animals alone. These results indicate that depletion of the NAC engages a stress response in the absence of HSF-1that can partially rescue the movement of worms under heat stress.

### ICD-1-depleted embryos contain enlarged lipofuscin granules

**Figure 7:**
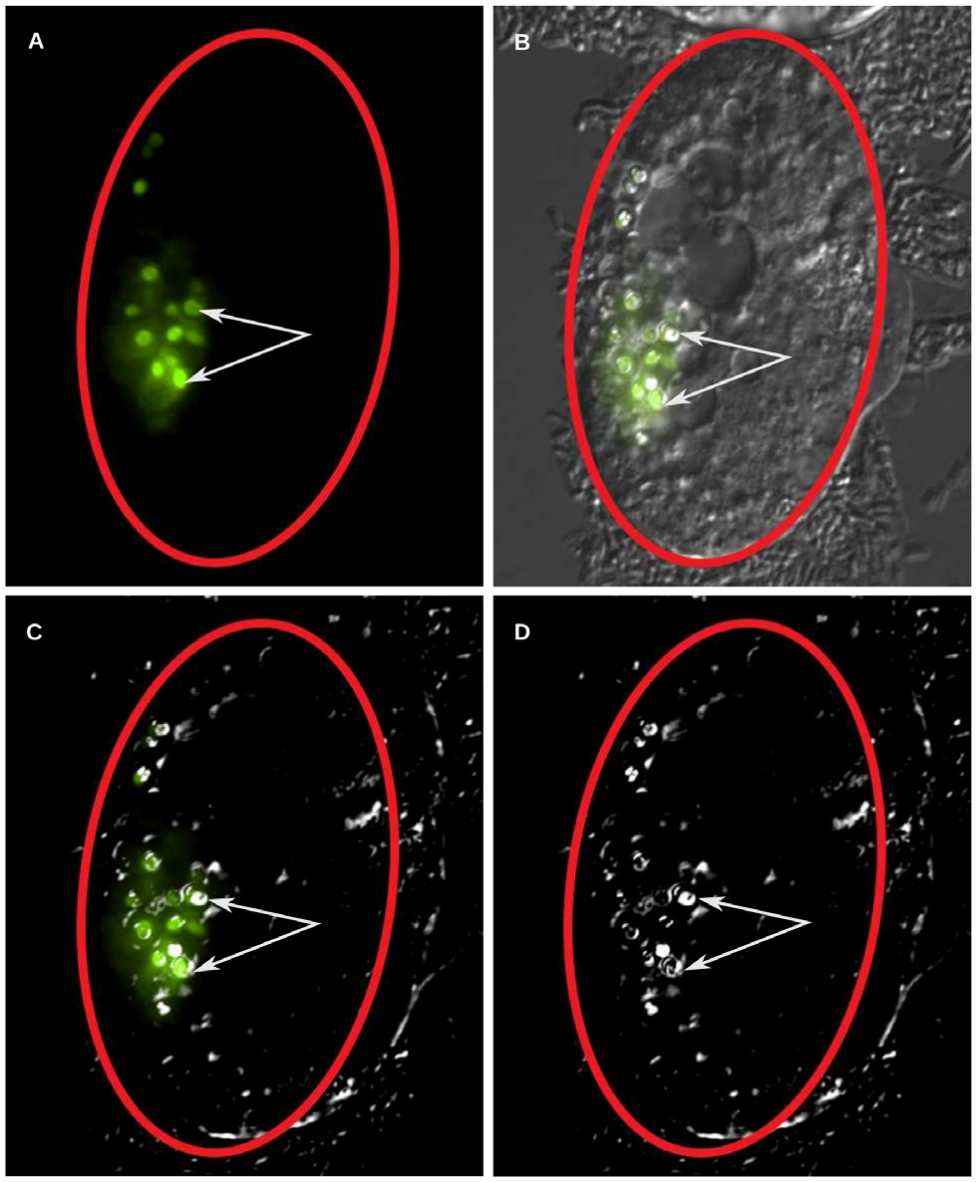
Generation of GLO-1-positive lysosomal structures in *icd-1*(RNAi) embryos. Animals containing a *glo-1*::GFP expression vector were treated with *icd-1*(RNAi) for 48–56 hours and the resulting embryos were scored for the presence of large lysosomal structures in their gut cells. GLO-1 is a marker for *C. elegans* lysosomes that are also distinguishable by bio-fluorescence under polarized light. A majority of the embryos (n = 20) displayed *glo-1*::GFP signal coincident with lysosomal structures. A–D) A representative [*glo-1*::GFP] *icd-1*(RNAi) embryo visualized by A) fluorescent light; B) fluorescent light and DIC; C) fluorescent light and polarized DIC and D) polarized DIC. White arrows indicate lysosomes visualized by DIC with overlapping *glo-1*::GFP signal, red ovals outline the shell of the embryo.

During the later stages of *icd-1*(RNAi) treatment, both wild type and *xbp-1*(ko) embryos contained abnormally high levels of corpse-like structures, a phenotype previously observed in ICD-1-depleted embryos [21]. We also observed in both populations large, bi-refringent, donut-like structures in the gut region of embryos that have been previously described as lysosomal organelles [30]. These structures were also observed in untreated embryos, but they were fewer in number and smaller in size (data not shown). We confirmed these enlarged granules were lysosomal in nature using a *glo-1*::GFP reporter strain. GLO-1 is a RAB GTPase expressed in the gut and a known marker of lysosome-related gut cell granules [30]. In these embryos, *glo-1*::GFP expression correlated with the bi-refringent granules observed in the DIC channel (Figure 7A–D).

**Figure 8:**
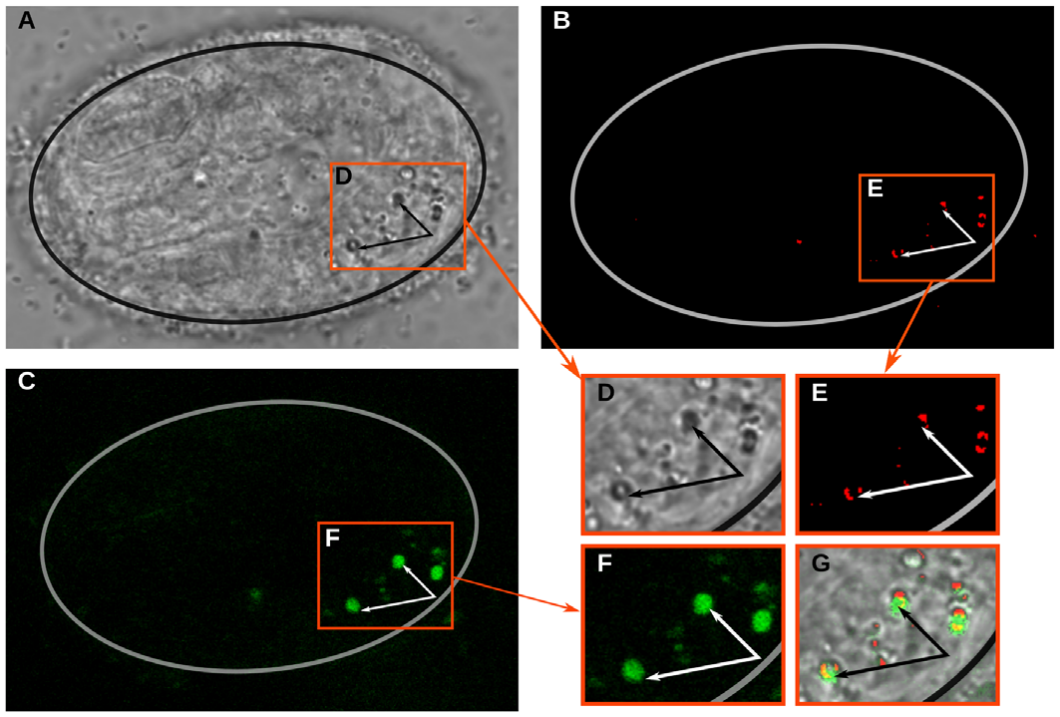
Identification of lipofuscin granules in *icd-1*(RNAi) embryos. Wild-type embryos were treated with *icd-1*(RNAi) and scored for the presence of lipofuscin granules using two-photon microscopy. A–C) a representative *icd-1*(RNAi) embryo visualized by A) transmission; B) thresholded transmission and; C) two-photon auto-fluorescence. Transmission microscopy was used to visualize large lysosomal structures in the gut cells, thresholded transmission used to visualize saturated pixels that correlated with lysosomal structures and two-photon excitation used to visualize specifically lipofuscin granules using a 500 nm bandwidth filter. D–F) magnified regions of A–C respectively, as demarcated by a red border. G) An overlay of images D–F. Arrows point to auto-flourescent lipofuscin granules.

Due to their bi-refringent properties and gut localization, we hypothesized that the enlarged lysosomal structures were specifically lipofuscin granules, as described by Clokely et. al (Figure 8D) [31]. Lipofuscins contain a mixture of aggregated proteins and lipids that are formed from damaged organelles and proteins that improperly degrade at the lysosome. Lipofuscin is known to generate a unique auto-fluorescent signal likely due to the presence of Schiff bases [23]. To confirm the presence of lipofuscin granules, we examined their auto-fluorescent properties using two-photon microscopy. Following the methods described by Yen et. al, we observed two-photon induced auto-fluorescence that localized to the enlarged, GLO-1-specific granules in *icd-1*(RNAi) embryos (Figure 8A,C, F,G) [32]. The lipofuscin granules were also identified in the transmission channel using binary contrast enhancement (thresholding) (Figure 8 B,E,G).

## Discussion

Here, we demonstrate the *C. elegans* NAC exhibits many characteristics of a translational chaperone. Depletion of either NAC subunit did not diminish the worm's viability in the presence of heat stress, and increased the worm's movement relative to wild-type worms, indicating a protective effect in the face of protein-denaturing stress. Depletion of ICD-1 up-regulated the expression of *hsp-4*, an ER chaperone and GRP78/BiP homologue associated with UPR function. Furthermore, the effects of *icd-1*(RNAi) were more pronounced in *xbp-1*(ko) animals, consistent with the loss of NAC inducing ER stress and the initiation of the UPR in wild-type worms. *hsf-1*(ko);*icd-1*(RNAi) animals were less resistant to heat stress than wild-type;*icd-1*(RNAi) animals, but considerably more robust than *hsf-1*(ko) worms exposed to heat, indicating that the stress response induced by ICD-1-depletion is partially dependent on the cytosolic stress sensor HSF-1, but other stress responders are able to rescue vitality in heat-stressed HSF-1(−) animals. Finally, we observed differential phenotypic effects of ICD-1-depletion corresponding to specific anatomical regions and cell types, indicating different cells and tissues may have unique stress responses to the loss of the NAC.

The NAC and its individual subunits have been assigned many roles since βNAC was first characterized as the transcription factor BTF3 in 1987 [33]. Recently, mounting evidence supports the NAC as a translational chaperone, a role previously hypothesized [1], [12]. In eukaryotes, the subunits of the NAC are the first two proteins to interact with the nascent polypeptide as it emerges from the ribosomal complex. In prokaryotes, trigger factor (TF) is the first protein to bind polypeptides, and it plays a vital role in *de novo* protein folding in bacteria [1], [14]. Despite its importance in proteostasis, TF knockouts show no growth defects due to the compensatory activity of redundant chaperones DNAk and DNAj, members of the HSP-70/40 family. Only when TF and chaperone DnaK are both deleted does lethality result. Similar to trigger factor, yeast NAC associates with both the ribosomal complex and the emerging polypeptide, but these complexes share no sequence homology, so it is not readily evident that they function similarly [14]. Recent discoveries, however, indicate that TF and the NAC are functionally related; yeast NAC knockouts show no growth defects until the translational chaperone Ssb, also a HSP-70/40 family member, is eliminated, indicating that, as with the loss of TF in bacteria, yeast may compensate for the loss of the NAC with redundant chaperone activity, protecting the organism from death [12].

If NAC(−) yeast maintain wild-type levels of growth by employing redundant chaperones, we wondered if *C. elegans* did the same. We hypothesized that in the absence of redundant chaperone activity, NAC depletion would induce toxic levels of misfolded protein, resulting in lethality. This hypothesis was based, in part, on previous findings showing gross morphological defects and increased cell death in the progeny of *icd-1*(RNAi) worms, and embryonic lethality in *icd-1* knockout mice, *Drosophila* and *C. elegans* [21], [34], [35]. These phenotypes are inconsistent with the presence of ICD-1-redundant activity; so it was surprising that both *icd-1* and *icd-2*(RNAi)-treated animals survived at least as well as wild-type worms under heat stress, and *icd-2*(RNAi)-treated worms appeared to survive at a higher rate. More striking were the differences in movement of ICD-1 or ICD-2-depleted animals relative to wild-type worms during heat stress, indicating that worms lacking the NAC were not just surviving but thriving in conditions that debilitated their wild-type counterparts. One explanation for this increase in vitality is the up-regulation of chaperones in NAC(−) animals that allow them to withstand heat stress more readily than NAC (+) worms. If so, NAC-depleted animals should show chaperone up-regulation. We observed this response in the form of increased *hsp-4* expression.

The up-regulation of *hsp-4* expression in NAC-depleted animals specifically implicates the generation of misfolded protein stress in the ER, and the initiation of the UPR as a consequence of NAC-depletion. HSP-4 is a homologue of GRP78/BiP, a chaperone and sensor of ER misfolded protein stress. As an ER-specific chaperone, GRP78/BiP helps refold misfolded proteins in this organelle, and also engages ER-specific stress sensors that carry out UPR functions [25], [26], [36]. An accumulation of misfolded proteins in the ER of NAC(−) animals is consistent with NAC's recently proposed role in yeast as a cotranslational chaperone that modulates the interaction of SRP with nascent polypeptides. Alamo *et al*. have shown that the NAC appears to associate with a vast majority of translating ribosomes and their polypeptides and this association allows the NAC to regulate polypeptide interactions with the SRP [11]. As such, the absence of the NAC may allow the SRP to bind to and localize a greater number of polypeptides to the ER, including those not normally targeted there, resulting in an overload of ER protein folding systems and misfolded protein stress. As earlier discussed, NAC-deficient yeast appear to maintain viability via the activities of chaperones that are redundant for NAC function and/or otherwise aid in protein folding processes. In *C. elegans*, the increase of *hsp-4* expression may serve the same purposes, through its own chaperone function and/or the up-regulation of other chaperones via initiation of the UPR.

The increased sensitivity of *xbp-1*(ko) animals to *icd-1*(RNAi) suggests that the loss of the NAC engages the Ire-1 receptor of the UPR. Ire-1 induces stress responses through two separate pathways that activate XBP-1 and Jun kinase (JNK) [16]. XBP-1 is a transcription factor that up-regulates chaperone expression and the ER-associated protein degradation system (ERAD), while JNK controls the induction of autophagy and apoptosis in response to the accumulation of misfolded protein. If loss of the NAC initiates Ire-1 signaling, as our data indicates, *xbp-1*(ko) animals would lack the increased chaperone activity and accelerated protein turnover provided by a functional XBP-1, but still be able to initiate apoptosis via the functional JNK pathway. In this context, *xbp-1*(ko);*icd-1*(RNAi) animals would accumulate misfolded protein rapidly relative to wild-type worms and more readily initiate apoptosis, consistent with our observations that *xbp-1*(ko);*icd-1*(RNAi) animals showed increased rates of cell death and overall mortality. The increased sensitivity of *xbp-1*(ko) worms in the absence of the NAC also indicates that the other arms of the *C. elegans* UPR. i.e. ATF6 and Perk/Pek-1, may provide partial, but not complete, redundancy for XBP-1. *xbp-1*(ko);*icd-1*(RNAi) animals up-regulated *hsp-4* relative to untreated controls, but not to the level of wild-type;*icd-1*(RNAi) worms, consistent with previous results that show up-regulation of *hsp-4* in *C. elegans* in response to the depletion of chaperones is reliant on XBP-1 [22].

The engagement of the ER-associated UPR in NAC(−) animals doesn't preclude contributions from other stress sensing pathways in response to NAC-depletion, particularly in light of the possibility that the NAC controls the localization of proteins throughout the cell. We found a vast majority of *hsf-1*(ko) animals immobile in the presence of heat, consistent with its role up-regulating cytosolic chaperones during stress, but these worms became dramatically more mobile when depleted of ICD-1, revealing a robust stress response in the absence of HSF-1 [37]. *hsf-1*(ko);*icd-1*(RNAi) animals were, however, still less mobile than wild-type;*icd-1*(RNAi) worms exposed to heat, so HSF-1 may contribute a portion of the stress response triggered by the absence of NAC. These results suggest animals with a complete UPR and functional HSF-1 are less sensitive to the loss of the NAC than animals missing either stress response element. If so, these results may indicate an accumulation of misfolded protein in both the ER and the cytosol of NAC(−) animals, inducing the activities of the UPR and HSF-1. Again, these results are consistent with previous findings by Alamo *et al*. that suggest the NAC controls the localization of proteins throughout the cell.

In the process of identifying a general cell stress response to the loss of the NAC, we also noticed potential cell-type specific responses to NAC depletion. Tissue- and cell-specific responses to misfolded protein stress can range from pathways engaged specifically in response to exogenous stress to those that are essential for normal differentiation [38]. In *C. elegans*, Kern *et al*. have identified stress responses to heat that differ significantly between neurons and muscle cells [39]. Specifically, they found that newly formed neurons under heat stress showed delayed up-regulation of chaperone expression and increased protein aggregations relative to young muscle cells, which exhibited a robust stress response. In our studies, the highly reproducible lack of *hsp-4* up-regulation in neuronal regions of *icd-1*(RNAi) embryos may explain why neurons are more sensitive to ICD-1 depletion, as measured by increases in cell death, relative to other cell types. This result is consistent with the findings of Kern *et al*. that show suboptimal chaperone expression in neurons undergoing misfolded protein stress. On the other hand, regions of the embryo containing gut and epidermal cells consistently displayed strong *hsp-4* up-regulation, and gut cells specifically showed a unique stress response to the loss of the NAC, the generation of large lysosomal structures we've putatively identified as lipofuscin granules.

The generation of lipofuscin granules in the gut cells of NAC(−) animals may causally link these structures to UPR-mediated autophagy, a process that shuttles misfolded proteins from the ER to lysosomal structures to relieve overburdened ERAD [40]. Lipofuscins are a chemical by-product of the incomplete degradation of proteins and lipids at the lysosome, forming a polymeric non-degradable substance that exhibits unique auto-fluorescent properties [23]. Since cellular materials are brought to the lysosome via autophagic mechanisms, lipofuscin formation necessarily depends on some type of autophagy. UPR-mediated autophagy in mammalian cells is dependent on the expression of GRP78/BiP, and previous studies have observed abnormal lysosomal formation in *C. elegans* under chemically induced ER stress. We observed large lipofuscin granules in NAC(−) gut cells presumably undergoing misfolded protein stress, resulting in the up-regulation of *hsp-4* and the engagement of the UPR; we postulate these lipofuscin granules are the direct result of UPR-mediated autophagy shuttling misfolded proteins, and perhaps damaged ER fragments, to these lysosomal structures.

We have presented evidence that *C. elegans* NAC displays many characteristics of a translational chaperone whose absence triggers the UPR, resulting in the up-regulation of chaperone expression, apoptosis and gut cell lipofuscins. Many questions concerning *C. elegans* NAC remain, including the scope of the stress response initiated by the depletion of the NAC, what aspects of the UPR are engaged, and what other stress responses might be contributing. Also of great interest are the activities of one NAC subunit in the absence of the other. Both subunits have been assigned individual functions, so it is likely that depletion of one subunit frees the other to affect cellular function in some way [10]. We cannot fully understand the outcomes resulting from the loss of one subunit without understanding how the remaining subunit functions. Ultimately, our understanding of NAC function under normal conditions and during times of stress is essential to our understanding of proteostasis, and will likely provide valuable insights into the pathology of diseases associated with aberrant protein folding and aggregation, particularly neurodegeneration. αNAC is down-regulated in Alzheimer's disease, and recent research has shown that exogenous amyloid aggregates expressed in human cells preferentially associate with a subproteome, including proteins involved in proteostasis; NAC was found in these aggregates [41], [42]. Using the genetically tractable and well characterized model of *C. elegans*, valuable insights into metazoan NAC function will continue to be gained. These insights will contribute significantly to our understanding of the human NAC and its roles in proteostasis in healthy and stressed cells.

## Materials and Methods

### Nematode strains

*C. elegans* strains were maintained at 20°C by standard methods [43]. N2 (wild-type), *hsp-4*::GFP (SJ4005), *hsp-16*::GFP (CL2070), *hsp-6*::GFP (SJ4100), *hsp-60*::GFP (SJ4058), [*hsp-4*::GFP] *xbp-1*(ko) (SJ17), *hsf-1*(ko) (PS3551), and *glo-1*::GFP (VS17) were all provided by the *Caenorhabditis* Genetics Center.

### RNA interference

RNA interference was performed by feeding *C. elegans* double stranded RNA correlating to the target genes of *icd-1* or *icd-2*, or the *unc-22* control. Feeder bacteria were obtained from Addgene, and grown from single colonies in liquid LB by shaking overnight at 37°C. During the last 4 hours of shaking IPTG was added to the overnight culture to a final concentration of 20 uM. After overnight shaking, cultured feeder was seeded onto RNAi plates. RNAi plates were made by combining 4.5 g NaCl, 25.5 g agar, 3.75 g peptone, 1.5 mL of 2 mg/mL uracil, 0.22 g CaCl, 0.75 g of 10 mg/ml cholesterol in 1463 mL of dH2O. This mixture was autoclaved, allowed to cool, and 37.5 mL of phosphate buffer (pH 6), 1.5 mL of 1 M MgSO4, 15 uL of 0.1 M IPTG and 1.5 mL of 25 mg/mL carbenicillin were added. Seeded plates were dried in an incubator for 24 hours at 37°C.

### Mobility and survival assays during heat stress

Gravid adult wild type worms were synchronized by bleaching worms in 1∶1 NaOH and bleach. Worms were bleached in 10 uL of lysis solution on the perimeter of medium sized OP50 plates. 28–30 hrs later synchronized F1 worms were moved to their respective experimental plates. Worms were moved to 3 plate types: 1) *icd-1*(RNAi) 2) *icd-2*(RNAi) or 3) OP50 bacteria. 50 worms were moved to each plate type. Plate types were made in duplicate in order to have temperature controls maintained at 20°C. All worm populations were grown at 20°C for 30 hours. After 30 hours of growth, heat shock populations were moved to a 36°C incubator. Worms were heat shocked continuously for 6 hours. Sampling began after 6 hours of heat exposure since behavioral heat shock effects are not measurable until then [29]. After 6 hours of heat shock, worms were periodically removed from the incubator and prodded with a platinum pick to examine mobility or survival. Responses were recorded as movement/survival or lack of movement/survival. Worms were sampled every hour. Movement was measured as displacement or escape reaction to physical prodding. Survival was measured as the presence of pharyngeal pumping after stimulus.

### Monitoring chaperone reporters during *icd-1*(RNAi)

L4s from the following strains SJ4005, CL2070, SJ4100 and SJ4058 were moved to *icd-1*(RNAi)or OP50 control plates. Worms were grown at 20°C for 48 hours. Adults were transferred to fresh plates after 24 hours to differentiate newly hatched embryos. After 48 hours, randomly chosen embryos were mounted on glass slides and viewed with a Nikon C1 confocal microscope for fluorescence.

### Quantification of *hsp-4*::GFP signal

Fluorescent images of embryos expressing *hsp-4*::GFP were captured with a Nikon C1 confocal microscope. Images were captured using brightfield settings and analyzed with NIS Elements software. Fluorescent intensity was measured with the line-scan measurement function. The line-scan measurement tool was centered and oriented along the long-axis of embryos. This measurement outputs fluorescent intensity (in arbitrary units) for each pixel in the line-scan. Data was exported into Microsoft Excel and the average intensity was calculated for OP50 and *icd-1*(RNAi) treatments. After random selection, 16 wild type and 13 *xbp-1*(ko) embryos were scanned and quantified.

### Lifespan assay

Pretzel-staged wild type and [*hsp-4*::GFP] *xbp-1*(ko) embryos were moved to *icd-1*(RNAi) plates and the percent survival of worms was determined daily. Survival was measured by prodding worms manually with a platinum pick and viewing for pharyngeal pumping. To determine baseline viability, control *xbp-1*(ko) and wild type strains were grown on OP50. Controls remained viable throughout the measurement period. Error bars represent the variation between two independent experiments n = 30.

### Embryo imaging

Embryo images (Figures 3, 4, and 5) were imported into GIMP image free ware. GFP and DIC images were overlain and embryo outlines were cropped along the embryo shell using the intelligent edge cropping function in GIMP. Image panels were compiled in Inkscape vector image free ware. Apoptotic cells and embryo shells were identified manually and highlighted in Inkscape (red or black outlines).

### Profiling of [*hsp-4*::GFP] *xbp-1*(ko) embryos

[*hsp-4*::GFP] *xbp-1*(ko) L4s were transferred to OP50 and *icd-1*(RNAi) plates. Embryos were monitored under the microscope after 48 hours of feeding. Embryo profiles were generated by classifying embryos morphologically at 100× magnification using DIC optics as early, mid, late or defective. The stage of wild-type embryos was determined primarily by the extent of the vermiform structure, e.g. early embryos showed no tube-like formation, while late embryos contained fully formed worms. Defective embryos were morphologically abnormal with high levels of cell death. Error bars represent the standard deviation of two independent experiments.

### Assessment of embryonic apoptosis

Cell corpses in embryos at the comma stage of embryogenesis were quantified using DIC optics as described [44].

### Lysosome imaging

To determine the identity of the bi-refringent objects in *icd-1*(RNAi) embryos, *glo-1*::GFP reporter worms were grown on feeder plates for 48 hours and examined. Morphologically defective embyros were mounted on glass slides and viewed with DIC and widefield flourescent optics using a Nikon C1 Confocal microscope. DIC and GFP flourescent channels were overlaid with NIS elements software. Polarized DIC images were captured by setting the polarizer to extinction.

### Two-Photon imaging and analyses

Embryos from worms fed *icd-1*(RNAi) for 48 hrs were mounted on agar pads in M9 solution, sealed with nail polish remover and imaged with a Zeiss 510 Meta System coupled to a Axioplan 200 M epifluorescence microscope within two hours of mounting. Embryos were viewed with a 63× Oil objective in light transmission and TPEF channels. The TPEF signal was captured with a 500/86 nm bandpass filter and detected with a photomultiplier tube. LSM images were imported into Image J using LSM Reader and LSM Toolbox. Dual channel images were separated with the stacks to images function and the pixel window was adjusted to 160 to remove background noise in the TPEF (green) channel.

## Supporting Information

Figure S1 **Embryonic expression of** ***hsp-4*** **in** ***xbp-1*** **(ko) animals depleted of ICD-1.** [*hsp-4*::GFP] *xbp-1*(ko) animals were fed *icd-1*(RNAi)-expressing bacteria or OP50 (*E. coli*) bacteria expressing no double stranded RNA for 36 hours and their progeny embryos were randomly assessed for expression of GFP. Two independent experiments displayed *hsp-4*::GFP up-regulation in the experimental population, one was quantified for levels of GFP expression relative to the control population. A) Average GFP signal of [*hsp-4*::GFP] *xbp-1*(ko) embryos treated with *icd-1*(RNAi) compared with GFP signal generated in control embryos (n = 8 for each population). Populations of embryos were chosen at random. Error bars represent the standard deviation of the mean GFP intensity of embryos. B) DIC and GFP overlapped images of representative [*hsp-4*::GFP] *xbp-1*(ko) control embryos (inset) or treated with *icd-1*(RNAi).
(TIF)
