# Supplementary figures and images for: Depletion of the C. elegans NAC Engages the Unfolded Protein Response, Resulting in Increased Chaperone Expression and Apoptosis
Source: PLoS One. 2012 Sep 5;7(9):e44038. doi: 10.1371/journal.pone.0044038 (PMC3434205; doi:10.1371/journal.pone.0044038)

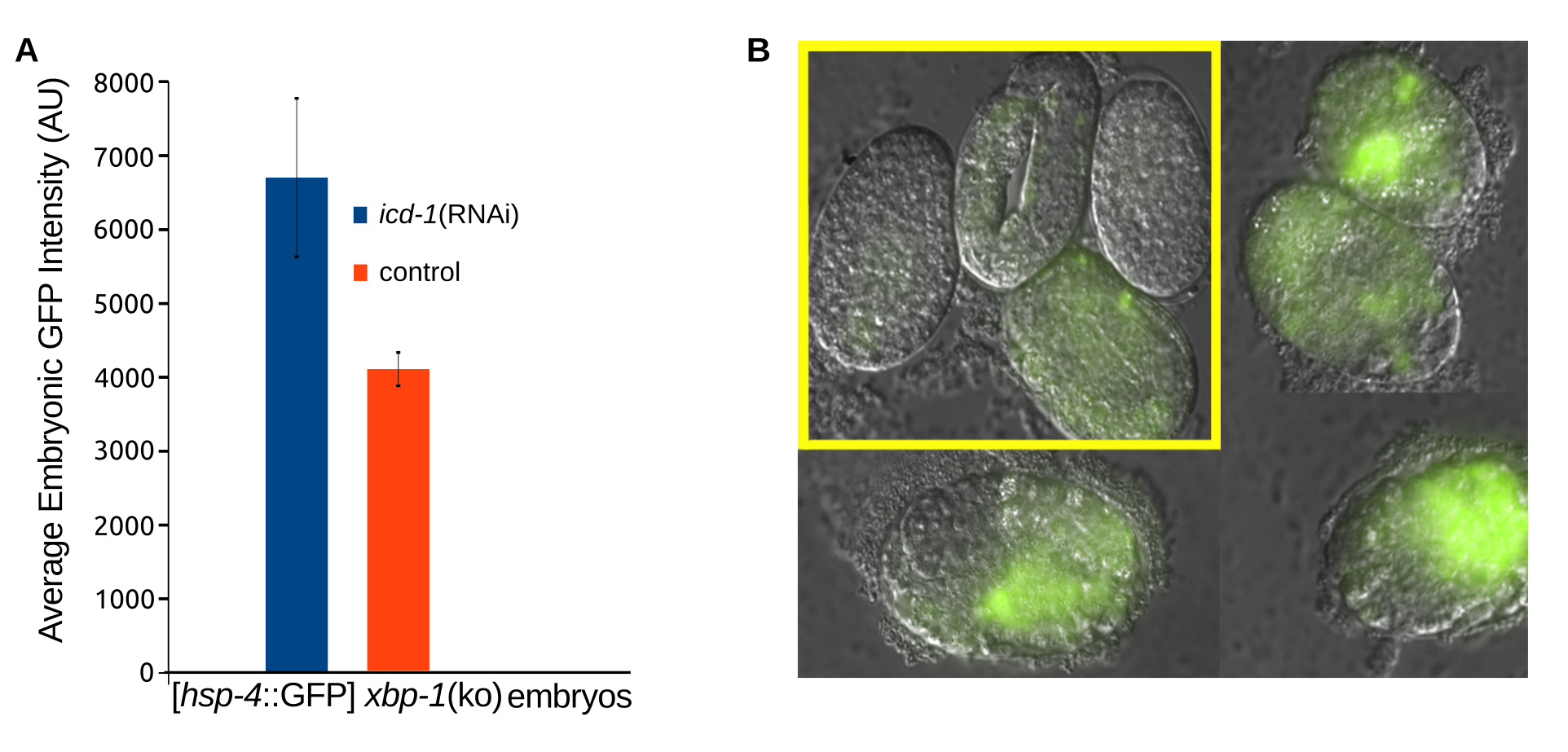

Supplement: Figure S1 — Embryonic expression of hsp-4 in xbp-1 (ko) animals depleted of ICD-1. [hsp-4::GFP] xbp-1(ko) animals were fed icd-1(RNAi)-expressing bacteria or OP50 (E. coli) bacteria expressing no double stranded RNA for 36 hours and their progeny embryos were randomly assessed for expression of GFP. Two independent experiments displayed hsp-4::GFP up-regulation in the experimental population, one was quantified for levels of GFP expression relative to the control population. A) Average GFP signal of [hsp-4::GFP] xbp-1(ko) embryos treated with icd-1(RNAi) compared with GFP signal generated in control embryos (n = 8 for each population). Populations of embryos were chosen at random. Error bars represent the standard deviation of the mean GFP intensity of embryos. B) DIC and GFP overlapped images of representative [hsp-4::GFP] xbp-1(ko) control embryos (inset) or treated with icd-1(RNAi). (TIF) [file pone.0044038.s001.tif]
